# Reduced DNAJC3 Expression Affects Protein Translocation across the ER Membrane and Attenuates the Down-Modulating Effect of the Translocation Inhibitor Cyclotriazadisulfonamide

**DOI:** 10.3390/ijms23020584

**Published:** 2022-01-06

**Authors:** Eva Pauwels, Becky Provinciael, Anita Camps, Enno Hartmann, Kurt Vermeire

**Affiliations:** 1 KU Leuven, Department of Microbiology, Immunology and Transplantation, Rega Institute for Medical Research, Laboratory of Virology and Chemotherapy, B-3000 Leuven, Belgium; eva.pauwels@kuleuven.be (E.P.); becky.provinciael@kuleuven.be (B.P.); anita.camps@kuleuven.be (A.C.); 2 Centre for Structural and Cell Biology in Medicine, Institute of Biology, University of Lübeck, 23562 Lübeck, Germany; enno.hartmann@uni-luebeck.de

**Keywords:** co-translational translocation, endoplasmic reticulum, cyclotriazadisulfonamide, ER quality control, DNAJC3, signal peptide, preprotein, Sec61 translocon, ribosome stalling

## Abstract

One of the reported substrates for the endoplasmic reticulum (ER) translocation inhibitor cyclotriazadisulfonamide (CADA) is DNAJC3, a chaperone of the unfolded protein response during ER stress. In this study, we investigated the impact of altered DNAJC3 protein levels on the inhibitory activity of CADA. By comparing WT DNAJC3 with a CADA-resistant DNAJC3 mutant, we observed the enhanced sensitivity of human CD4, PTK7 and ERLEC1 for CADA when DNAJC3 was expressed at high levels. Combined treatment of CADA with a proteasome inhibitor resulted in synergistic inhibition of protein translocation and in the rescue of a small preprotein fraction, which presumably corresponds to the CADA affected protein fraction that is stalled at the Sec61 translocon. We demonstrate that DNAJC3 enhances the protein translation of a reporter protein that is expressed downstream of the CADA-stalled substrate, suggesting that DNAJC3 promotes the clearance of the clogged translocon. We propose a model in which a reduced DNAJC3 level by CADA slows down the clearance of CADA-stalled substrates. This results in higher residual translocation into the ER lumen due to the longer dwelling time of the temporarily stalled substrates in the translocon. Thus, by directly reducing DNAJC3 protein levels, CADA attenuates its net down-modulating effect on its substrates.

## 1. Introduction

In eukaryotic cells, protein translocation into the endoplasmic reticulum (ER) is the first and decisive step in the biogenesis of secretory and integral membrane proteins [1,2,3,4,5,6]. This translocation process is typically guided by protein-specific signal peptides (SPs) and their interplay with the different components of the translocation machinery that are present in the cytosol, the ER membrane and the ER lumen [1,2,3,4,5,6].

Eukaryotic ER protein translocation is mediated by the heterotrimeric Sec61 translocon. The Sec61 translocon consists of α, β and γ monomers that together form an aqueous pore that spans the ER membrane [7,8,9,10]. Depending on the overall size of the precursor protein and its hydrophobicity and/or amino acid content, ER protein translocation occurs post- or co-translationally [11,12,13,14]. In higher eukaryotes, co-translational protein translocation is the most common and couples protein translation directly to its translocation over the ER membrane [11,14,15]. The interaction of the SP with the Sec61 translocon results in conformational changes of the translocon that eventually lead to the translocation of the preprotein into the ER lumen [7,9,16,17]. In the case of integral membrane proteins, the hydrophobic transmembrane domain (TMD) is integrated into the ER membrane via lateral movement from the translocon, mediated by the lateral gate of the Sec61 translocon [2,7]. Once preproteins are co-translationally translocated into the ER lumen, they are post-translationally modified (i.e., SP cleavage by the signal peptidase complex and protein glycosylation by the oligosaccharyl transferase complex), folded and transported to the Golgi apparatus for further maturation [2,3,17,18,19,20].

The accumulation of mislocalised, misfolded and/or malfunctioning proteins due to dysfunctional protein processing (i.e., protein post-translational modifications, folding and/or assembly in the ER lumen), triggers ER stress, which is detrimental for overall cellular functions. Cells have, therefore, acquired sophisticated ER quality control processes such as the unfolded protein response (UPR) [21,22,23,24]. Three ER stress sensors, i.e., activating transcription factor 6 (ATF6), inositol requiring enzyme 1 (IRE1) and PKR-like endoplasmic reticulum kinase (PERK) activate the UPR via the transcriptional upregulation of molecular chaperones to refold misfolded proteins in the ER lumen and inhibit global protein synthesis to reduce the load of client proteins, while apoptotic pathways are activated to eliminate severely damaged cells [21,22,23,25,26,27,28,29,30,31].

During ER stress, DnaJ Homolog Subfamily C member 3 (DNAJC3, also known as ERdj6 or p58^IPK^) plays a role in the UPR to restore ER homeostasis [32,33,34]. After its transcriptional upregulation via ATF6 and IRE1, DNAJC3 is co-translationally translocated into the ER lumen where it functions as the co-chaperone alongside the binding immunoglobulin protein (BiP) to refold misfolded proteins in the ER lumen [32,33,35]. Once the lumenal proteins are properly folded and ER stress is (at least partly) relieved, DNAJC3 is involved in the activation of protein synthesis via the inhibition of PERK, thus, indirectly enhancing the initiation of protein translation [31,36,37]. Hence, DNAJC3 helps to restore protein levels and, therefore, reassures cellular processes post ER stress.

As stated earlier, ER stress is undoubtedly detrimental for cells, and, in general, contributes to the pathogenesis of many, mostly neurological, diseases [38,39,40,41]. Mutations in BiP co-chaperones and proteins involved in UPR have been associated with nervous system abnormalities [39,42,43,44,45,46,47,48,49]. Furthermore, DNAJC3 loss-of-function mutations have been identified in patients that were diagnosed with early-onset diabetes mellitus and suffered from multisystemic neurodegeneration (including ataxia, sensorimotor neuropathy and sensorineural hearing loss), which appear to be systematic features of the DNAJC3-diseased phenotype [50,51,52]. Studies highlight the importance of cholesterol homeostasis and lipid metabolism at the ER to underly the pathophysiology of DNAJC3 loss-of-function mutations. DNAJC3-deficient cells were presented with accumulated levels of cholesterol and lipids at the ER membrane [39,53,54], which disrupted its composition and overall structure and, thus, triggered ER stress [39,55,56,57,58,59]. Altogether, it is clear that DNAJC3 provides a multifunctional role in protecting cells during ER stress.

As well as folding-related ER stress, evidence has shown that erroneously halted protein translocation also induces considerable levels of ER stress, as the partially translocated polypeptide clogs the translocon [24,60,61]. From this perspective, the retarding of protein translocation into the ER by specific Sec61 translocon inhibitors might also induce ER stress and activate cellular pathways to clear the clogged translocons. How cells cope with clogged translocons has since emerged as an intriguing question; however, little is known about the translocon-associated quality control processes to safeguard protein translocation [24].

**Figure 1:**
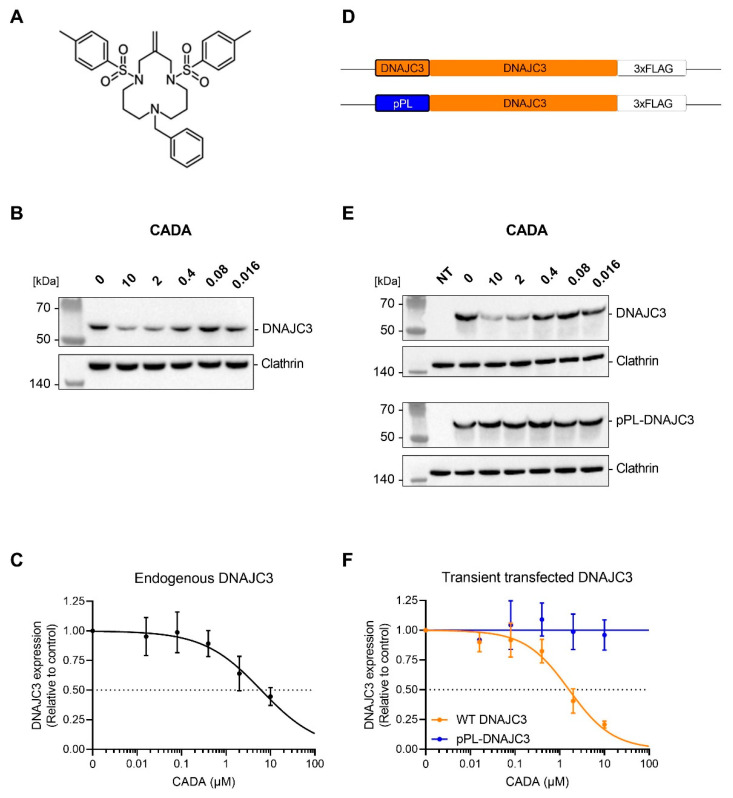
CADA sensitivity of endogenous and transfected DNAJC3 in HEK293T cells. (**A**) Chemical structure of CADA. (**B**) Western blot images of cell lysates from HEK293T cells treated for 24 h with different CADA concentrations. Protein bands were visualized with an antibody against DNAJC3 and an anti-clathrin antibody was used for the cell loading control. One representative experiment out of four is shown. (**C**) Concentration–response curves of CADA for endogenous DNAJC3 expression in HEK293T cells. Samples from (**B**) were quantified and normalised to the clathrin internal control. A four-parameter concentration–response curve was fitted to the data from four replicate experiments. Values are mean ± SD; *n* = 4. (**D**) Schematic representation of the DNAJC3 variants, with different signal peptides. (**E**) Western blot images of cell lysates from non-transfected (NT), DNAJC3-FLAG- or pPL-DNAJC3-FLAG-transfected HEK293T cells treated for 24 h with different CADA concentrations. Protein bands were visualized with an antibody against the FLAG tag and an anti-clathrin antibody was used for the cell loading control. One representative experiment out of five is shown. (**F**) Concentration–response curves of CADA for DNAJC3- and pPL-DNAJC3-transfected HEK293T cells. Samples from (**E**) were quantified and normalised to the clathrin internal control. A four-parameter concentration–response curve was fitted to the data from at least four replicate experiments. Values are mean ± SD; *n* ≥ 4. CADA: cyclotriazadisulfonamide; HEK293T: human embryonic kidney 293T cells; DNAJC3: DnaJ homolog subfamily C member 3; pPL: pre-prolactin.

CADA is a small synthetic macrocycle (Figure 1A) that was discovered during an anti-HIV screening program [62,63,64,65]. Previous work by our laboratory showed that CADA inhibits the co-translational translocation process of human CD4 (huCD4), a type I membrane protein, by direct interaction with the receptor’s SP, resulting in the downmodulation of huCD4 on the surface of cells and a significant reduced entry and replication of HIV in human cells [62,63,64,65,66]. Later, sortilin and 4-1BB were also identified as targets for CADA. A recent proteomic study conducted on the membranome of T-cells revealed only three additional substrates, i.e., ERLEC1, PTK7 and DNAJC3, evidencing high substrate specificity for the ER protein translocation inhibitor CADA [67,68,69,70].

Thus, (i) CADA inhibits ER translocation of a specific set of substrates, presumably resulting in (temporarily) clogged translocons that might cause ER stress, and (ii) DNAJC3 is a CADA substrate that should be normally upregulated during ER stress. Therefore, we questioned if restored DNAJC3 levels under CADA pressure could influence the inhibitory effect of CADA. We compared the sensitivity of substrates to CADA in the presence of WT DNAJC3 or a CADA-resistant DNAJC3 mutant protein and observed the enhanced sensitivity of huCD4, PTK7 and ERLEC1 for CADA when DNAJC3 was expressed at high levels.

## 2. Results

### 2.1. CADA Reduces Cellular Expression of DNAJC3

In a recent proteomics survey, DNAJC3 has been validated as a target protein for CADA [69]. The treatment of HEK293T cells with increasing concentrations of CADA results in a concentration-dependent reduction in endogenous DNAJC3 expression (Figure 1B,C), with profoundly reduced DNAJC3 levels at 2 and 10 µM of CADA (IC_50_ = 6.2 µM). Due to the way DNAJC3 functions in the unfolded protein response during ER stress, we questioned if the reduced expression of DNAJC3 by CADA could affect the ER translocation of other CADA substrates. Previous work has shown that the sensitivity of targets to CADA, such as huCD4, 4-1BB, SORT, ERLEC1, PTK7 and DNAJC3, is intrinsic to the cleavable SP and the N-terminal region of the mature protein [64,68,69]. By inserting the huCD4 SP and the first 7 amino acid residues of the mature huCD4 protein into the CADA-resistant preprolactin (pPL) protein, we can introduce sensitivity of pPL to CADA as determined by immunoblotting (Supplementary Figure S1A,B), as similarly described for the CADA-resistant murine CD4 (mCD4) [68]. Vice versa, exchanging the wild-type SP of CADA-sensitive proteins by that of pPL or mCD4 results in the generation of a CADA-resistant protein [64]. Thus, we generated a CADA-resistant DNAJC3 protein (designated as pPL-DNAJC3) by replacing the CADA-sensitive SP of DNAJC3 with the CADA-resistant SP of pPL (see Figure 1D). We also introduced a triple-FLAG sequence at the C-terminus of DNAJC3 for detection purposes. As expected, the transfection of HEK293T cells with the DNAJC3 plasmids resulted in high protein levels of DNAJC3 that outranged the endogenous expression of DNAJC3 (Supplementary Figure S1C,D). HEK293T cells transfected with WT DNAJC3 showed a concentration-dependent sensitivity of DNAJC3 to CADA (IC_50_ of 1.6 µM) (Figure 1E,F), which was slightly higher than that of endogenous DNAJC3 (Figure 1C). In contrast, the transfection of cells with the pPL-DNAJC3 construct resulted in a CADA-resistant phenotype with a constant expression of DNAJC3, as well as at the highest CADA concentrations tested (Figure 1F). Thus, the replacement of the DNAJC3 SP with the SP of pPL resulted in the complete loss of sensitivity of DNAJC3 to CADA.

### 2.2. CADA Inhibits the Signal Peptide Dependent Co-Translational Translocation of the ER Lumenal DNAJC3 Protein

**Figure 2:**
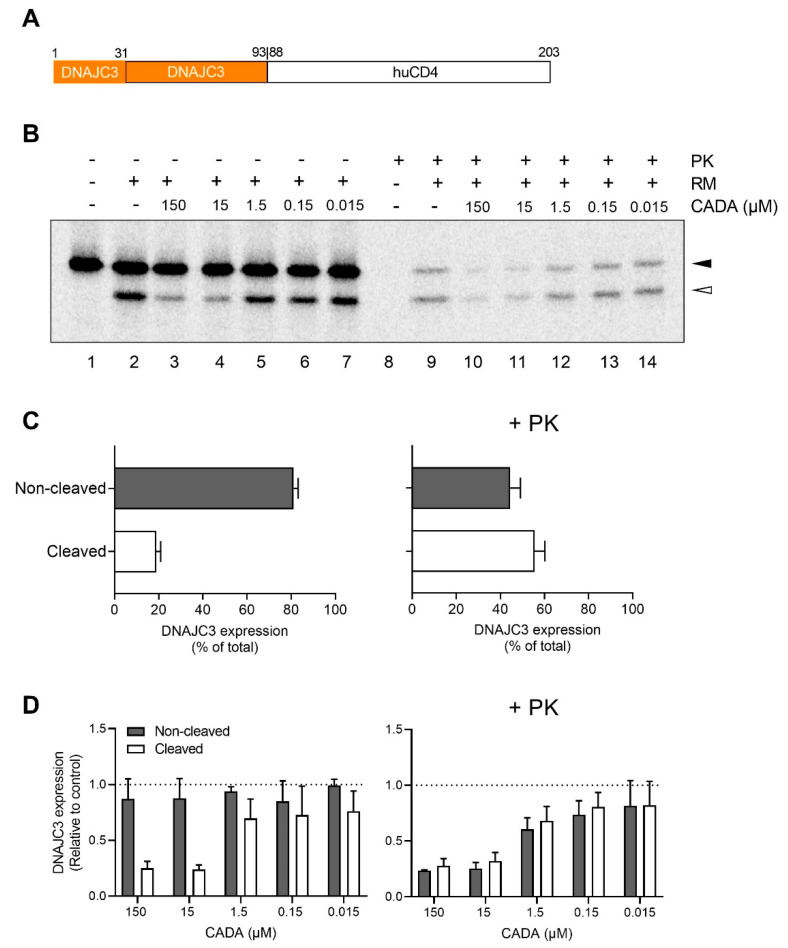
CADA inhibits the signal peptide-dependent co-translational translocation of the ER lumenal DNAJC3 protein. (**A**) Representation of the construct used for cell-free in vitro translation and translocation assay. (**B**) Cell-free in vitro translation and translocation in rabbit reticulocyte lysate supplemented with ovine microsomes, CADA and proteinase K (PK). Autoradiogram of the in vitro translated and translocated DNAJC3-huCD4 chimaeric protein. In the presence of rough microsomes (RM), the preprotein (black arrowhead) was translocated into the ER lumen and the SP was cleaved, resulting in a faster migrating mature protein (open arrowhead). One representative experiment out of three is shown. (**C**) Percentage of non-cleaved (black arrowhead, preprotein) and cleaved (open arrowhead, mature protein) DNAJC3 protein in the DMSO-treated control sample of untreated and PK-treated samples. Samples from (A, lane 2 and lane 9) were quantified. Bars are mean ± SE; *n* = 3. (**D**) CADA sensitivity of the non-cleaved and cleaved DNAJC3 protein fraction in control samples and PK-treated samples. Samples from A (lanes 2–7 and 9–14) were quantified and normalised to the respective protein fraction in the DMSO control (lane 2 or 9). Bars are mean ± SE; *n* = 3. CADA: cyclytriazadisulfonamide; DNAJC3: DnaJ homolog subfamily C member 3; PK: Proteinase K; RM: rough microsomes; ER: endoplasmic reticulum; SP: signal peptide; DMSO: dimethyl sulfoxide.

As the SP was cleaved from the DNAJC3 preprotein during ER translocation, we expected that both WT DNAJC3 and the chimaeric pPL-DNAJC3 constructs expressed the same mature DNAJC3 protein. To verify that DNAJC3 is translocated into the ER lumen, cell-free in vitro translation experiments were performed (Figure 2). As initial attempts to translate the full-length WT DNAJC3 protein were not successful (data not shown), we switched to an alternative DNAJC3/huCD4 chimaeric protein. This truncated soluble huCD4 construct contained the SP of DNAJC3 and the first 62 residues of mature DNAJC3 (see scheme in Figure 2A). In vitro translation of DNAJC3/huCD4 resulted in substantial levels of translated preprotein and detectable SP cleavage (Figure 2B, lane 2). Of note, more than one-third of the DNAJC3 species retained the SP after translocation as determined by proteinase K (PK) treatment (Figure 2C, right panel), an observation that is in line with another report [71] (Supplementary Figure S2). The translocated SP-containing DNAJC3 species were fully protected from PK treatment (Figure 2B, lanes 8–14), indicating that these proteins were localised in the lumen of the ER. Of course, for the translocated SP-containing DNAJC3 protein, the PK protection assay could not distinguish between membrane-anchored (by the SP) and luminal free-floating DNAJC3 species. Notably, CADA inhibited the translocation of both non-cleaved and cleaved DNAJC3 species in an equal manner (Figure 2B, lanes 9–14 and Figure 2D, right panel), whereas the preprotein levels in the control samples seemed to be unaffected by CADA (Figure 2B, lanes 2–7 and Figure 2D, left panel). In addition, DNAJC3 has also been proposed as an uncleaved type I transmembrane protein that inserts in the ER membrane in a head-on formation with the SP as the membrane anchor and with the majority of the protein exposed to the cytosol [71]. The relatively high amount of DNAJC3 preprotein as compared to the SP-cleaved species in the non-PK samples (Figure 2B, lanes 2–7 and Figure 2C, left panel), might suggest the appearance of such a membrane-anchored cytosolic protein. However, from these radioblots, we could not make a distinction between a free non-targeted preprotein and ER membrane-anchored DNAJC3 preprotein. Finally, DNAJC3 did not contain potential N-glycosylation sites, thus, excluding the possibility of tracking the translocated protein fraction by ER lumenal N-glycosylation.

### 2.3. Presence of Cellular DNAJC3 Enhances the Sensitivity of Substrates to CADA

**Figure 3:**
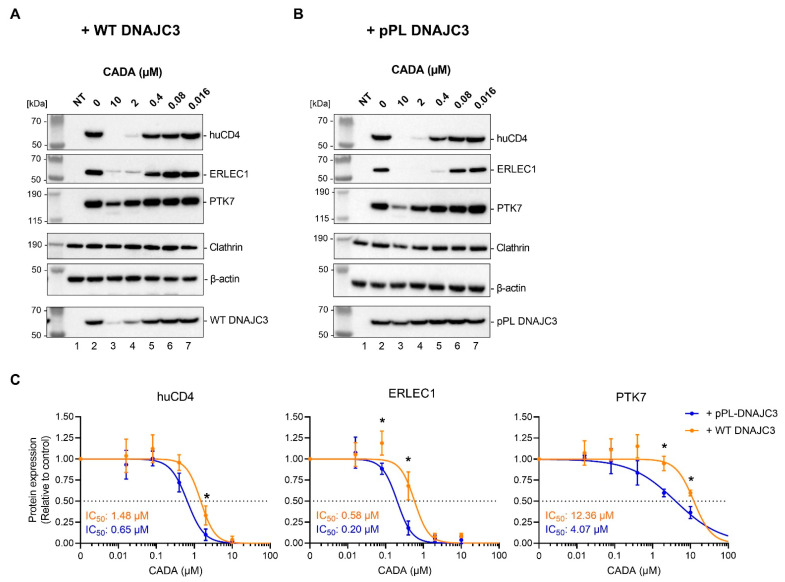
DNAJC3 sensitizes targets to CADA. (**A**) Western blot images of cell lysates from non-transfected (NT) HEK293T cells, and huCD4-V5, ERLEC1-V5 or PTK7-V5, co-transfected with WT DNAJC3. Cells were treated for 24 h with different CADA concentrations and subjected to immunoblotting. Protein bands were visualized with an antibody against the V5 tag, and an antibody against clathrin (huCD4 and ERLEC1) or β-actin (PTK7) was used for the cell loading controls. An antibody against the FLAG tag was used to detect the co-transfected WT DNAJC3 in the samples. One representative experiment out of three to six is shown. The clathrin loading control shown is that of the ERLEC1 sample. The respective loading control for huCD4 and WT DNAJC3 is presented in Supplementary Figure S3D. (**B**) Same as in (**A**) but for co-transfection with pPL-DNAJC3. One representative experiment out of three to six is shown. The clathrin loading control shown is that of the huCD4 sample. The respective loading control for ERLEC1 and pPL-DNAJC3 is presented in Supplementary Figure S3D. (**C**) Concentration–response curves of CADA for huCD4, ERLEC1 and PTK7 in transfected HEK293T cells. Samples from (**A**,**B**) were quantified and normalised to the clathrin (huCD4 and ERLEC1) or β-actin (PTK7) internal control. A four-parameter concentration–response curve was fitted to data from at least three replicate experiments. Values are mean ± SD; *n* ≥ 3. Statistical analysis (multiple unpaired *t*-tests) showed significantly decreased expression of huCD4, ERLEC1 and PTK7 when co-expressed with pPL-DNAJC3 as compared to WT DNAJC3 (* = *p* < 0.05). HEK293T: human embryonic kidney 293T cells; huCD4: human CD4; ERLEC1: endoplasmic reticulum lectin 1; PTK7: inactive tyrosine-protein kinase 7; pPL: pre-prolactin; DNAJC3: DnaJ homolog subfamily C member 3; CADA: cyclotriazadisulfonamide.

To analyze if, under CADA pressure, the reduced DNAJC3 levels can affect the sensitivity of proteins to CADA, we co-transfected HEK293T cells with a V5-tagged huCD4 construct and either the FLAG-tagged WT DNAJC3 plasmid or the CADA-resistant pPL-DNAJC3 plasmid. The transiently transfected cells were then treated with increasing concentrations of CADA and subjected to immunoblotting. Similar co-transfections were performed with the V5-tagged ERLEC1 or PTK7 constructs. Both huCD4 and PTK7 are type I integral membrane proteins mainly expressed at the cell surface, whereas ERLEC1 is a soluble protein that resides in the ER lumen. In accordance with our previous report, the expression of the three V5-tagged proteins was differentially affected by CADA [69] (Supplementary Figure S3A,B). As summarized in Figure 3A, in the non-treated control cells, transfection with the different plasmids resulted in the high expression level of the V5-tagged target proteins and the co-transfected FLAG-tagged DNAJC3 protein. Treatment with CADA concentration dependently reduced the protein expression of huCD4, ERLEC1 and PTK7 when WT DNAJC3 was expressed (Figure 3A,C). Interestingly, the co-expression of the target proteins with pPL-DNAJC3 resulted in a stronger CADA effect as compared to WT DNAJC3 co-expression. This was evidenced by the significantly lower protein levels detected (Figure 3B versus Figure 3A) and by the comparison of the corresponding calculated IC_50_ values (Figure 3C). Altogether, these data indicate that enhanced levels of DNAJC3 result in the sensitization of selective substrates to CADA.

### 2.4. DNAJC3 Enhances Proteasomal Degradation of CADA-Stalled PTK7 Preprotein

Previous experiments clearly indicate that the amount of translocated (mature) protein is more reduced by CADA when DNAJC3 is highly expressed. The lower levels of translocated protein induced by CADA treatment can be the net result of the stronger inhibition of protein translocation (lower entry efficiency into ER) or faster clearance of putative mis-targeted proteins from the ER by DNAJC3 (higher exit efficiency from ER). However, the role of DNAJC3 in this process is not entirely understood.

**Figure 4:**
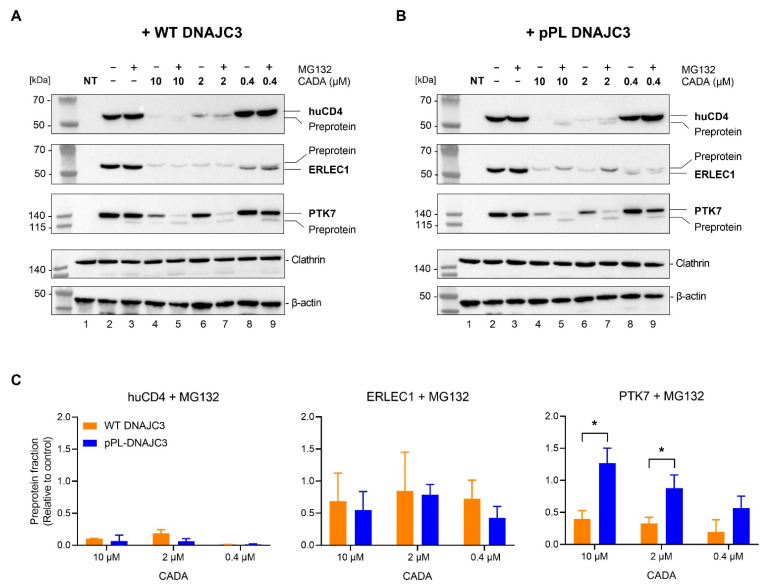
Inhibition of the proteasome in the presence of CADA rescues a preprotein fraction which is DNAJC3-dependent for PTK7. (**A**) Western blot images of cell lysates from non-transfected (NT) HEK293T cells, and huCD4-V5, ERLEC1-V5 or PTK7-V5, co-transfected with WT DNAJC3. Cells were treated for 24 h with different CADA concentrations and a constant dose of MG132 (200 nM). Protein bands were visualized with an antibody against the V5 tag, and an antibody against clathrin (huCD4 and ERLEC1) or β-actin (PTK7) was used for the cell loading controls. One representative experiment out of two to four is shown. The clathrin loading control shown is that of the ERLEC1 sample. The respective loading control for huCD4 is presented in Supplementary Figure S3E. (**B**) Same as in (**A**) but for co-transfection with pPL-DNAJC3. One representative experiment out of two to four is shown. (**C**) Preprotein fraction of MG132-treated samples quantified from (**A**,**B**) and normalised to the internal loading control of the respective sample. Bars are mean ± SE; *n* = 2 for huCD4 and PTK7; *n* = 4 for ERLEC1. Statistical analysis (multiple unpaired *t*-tests) showed increased detection of the PTK7 preprotein when co-expressed with pPL-DNAJC3 as compared to WT DNAJC3 (* = *p* < 0.05). HEK293T: human embryonic kidney 293T cells; huCD4: human CD4; ERLEC1: endoplasmic reticulum lectin 1; PTK7: inactive tyrosine-protein kinase 7; pPL: pre-prolactin; DNAJC3: DnaJ homolog subfamily C member 3; CADA: cyclotriazadisulfonamide; DMSO: dimethyl sulfoxide.

First, we addressed if the CADA inhibition of protein translocation is linked to the proteasomal degradation of the CADA substrates. Thus, we analyzed the effect of the proteasome inhibitor MG132 on the CADA samples. Briefly, we co-transfected HEK293T cells with V5-tagged huCD4, ERLEC1 or PTK7 and either the FLAG-tagged WT DNAJC3 plasmid or the CADA-resistant pPL-DNAJC3 plasmid. Cells were then treated with increasing concentrations of CADA in combination with a fixed dose of MG132 (200 nM) for 24 h and subjected to immunoblotting. As shown in Figure 4A, in the non-treated control cells, the expression of the substrates was not affected by treatment with the proteasome inhibitor MG132 only (Figure 4A, lanes 2 vs. 3). However, for PTK7, a faint lower band on the gel could be detected for the MG132-treated control sample (Figure 4A, lane 3) that corresponded to the non-glycosylated preprotein fraction as determined by Endo H treatment (Supplementary Figure S3C). The combined treatment of CADA with MG132 did result in the rescue of the preprotein, which was most evident for PTK7 (Figure 4A lanes 5, 7 and 9). Remarkably, the combination of 10 or 2 µM of CADA with MG132 completely abolished the expression of mature PTK7, demonstrating that MG132 has a synergistic effect on CADA in blocking the translocation of PTK7. Additionally, for huCD4, 2 µM CADA treatment in combination with MG132 further reduced the low level of mature (glycosylated) protein, as compared to CADA treatment only, and resulted in the rescue of the huCD4 preprotein, although at a nearly detectable and very low amount (Figure 4C). For ERLEC1, the complete inhibition of protein translocation was achieved with the higher concentrations of CADA. Here, the only fraction of ERLEC1 that could be (weakly) visualized was the slower migrating protein band that corresponds to the preprotein, given that ERLEC1 is an ER-resident protein that is not glycosylated (Supplementary Figure S3C), but of which the SP is cleaved upon translocation into the ER lumen. The addition of MG132 did not change the outcome of CADA treatment. This also suggests that, in contrast to huCD4 and PTK7, ERLEC1 has a more stable preprotein that is already visualised without the inhibition of the proteasome. In line with the data described above, the treatment of CADA alone resulted in the reduced expression of the mature protein fraction in a concentration-dependent manner that was more pronounced when the proteins were co-transfected with pPL-DNAJC3 as compared to co-transfection with WT DNAJC3 (Figure 4A,B, respectively). For ERLEC1, the absolute amount of rescued preprotein with MG132 remained little and somehow constant over the different CADA concentrations, irrespective of enhanced DNAJC3 expression (Figure 4C). Interestingly, when DNAJC3 was highly expressed (Figure 4B), the combined treatment of CADA with MG132 rescued proportionally more preprotein of PTK7 that reached significance for the 10 and 2 µM CADA samples (*p* = 0.034 and 0.047, respectively) (Figure 4C), indicating that DNAJC3 enhanced the proteasomal degradation of CADA-stalled PTK7 preprotein.

### 2.5. DNAJC3 Differentially Affects the Expression of Cytosolic or ER Translocated Proteins in the Presence of CADA-Stalled Substrates

**Figure 5:**
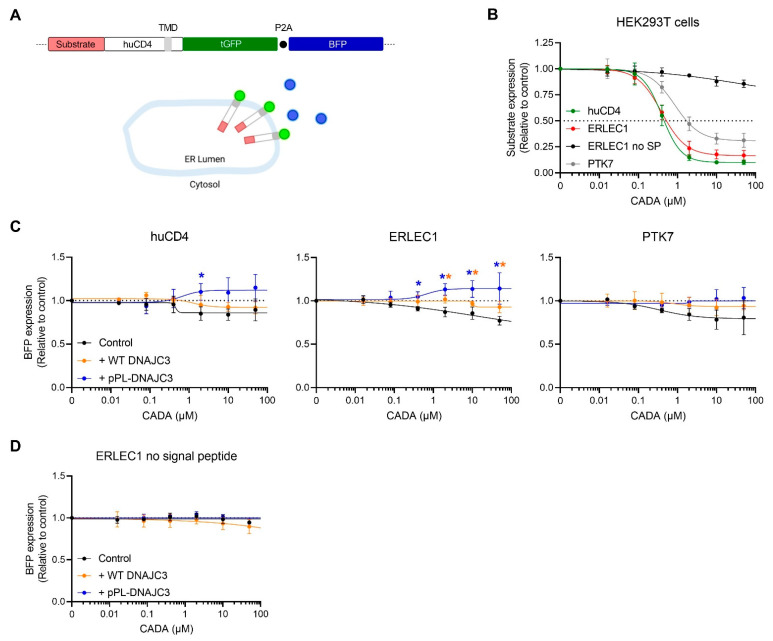
DNAJC3 differentially affected BFP expression in the presence of CADA-stalled proteins. (**A**) Representation of the tGFP-P2A-BFP construct. The construct expressed huCD4 that was anchored in the plasma membrane via its transmembrane domain (TMD) and with tGFP at the cytosolic tail. As the SP was cleaved by the ER lumenal signal peptidase during protein biogenesis, the mature huCD4 variants differed in only 62 amino acids at their N-terminus. (**B**) Four-parameter concentration–response curves for the CADA of huCD4, ERLEC1, ERLEC1 with no SP, and PTK7 cloned in the same tGFP-P2A-BFP plasmid backbone as shown in (**A**). HEK293T cells were transiently transfected with the tGFP-P2A-BFP constructs and incubated with different CADA concentrations for 24 h. Transfected tGFP-P2A-BFP plasmid DNA was equal to the transfected tGFP-P2A-BFP plasmid DNA in the co-transfected conditions of (**C**,**D**). Protein levels of tGFP (representing the level of substrate) in CADA-treated samples were normalised to the DMSO control (set at 1.00). Curves were fitted to data from three to five replicate experiments. Values are mean ± SD; *n* ≥ 3. (**C**) Four-parameter concentration–response curves for CADA of the BFP signal of huCD4, ERLEC1 and PTK7 cloned in the same tGFP-P2A-BFP plasmid backbone as shown in (**A**). HEK293T cells were transiently transfected with the tGFP-P2A-BFP construct, or co-transfected with the tGFP-P2A-BFP construct and WT DNAJC3 or pPL-DNAJC3 and incubated with different CADA concentrations for 24 h. Transfected tGFP-P2A-BFP plasmid DNA was equal to the transfected tGFP-P2A-BFP plasmid DNA in the co-transfected conditions. BFP levels in CADA-treated samples were normalised to the DMSO control (set at 1.0). Curves were fitted to data from three to five replicate experiments. Values are mean ± SD; *n* ≥ 3. Statistical analysis (multiple unpaired *t*-tests) showed significantly increased expression of BFP for huCD4 and ERLEC1 when co-expressed with pPL-DNAJC3 and for ERLEC1 when co-expressed with WT DNAJC3 as compared to the control (* = *p* < 0.05). (**D**) Same as in (**C**). Four-parameter concentration–response curve for the CADA of the BFP signal of ERLEC1 with no SP cloned in the tGFP-P2A-BFP plasmid backbone. Curves were fitted to data from three replicate experiments. Values are mean ± SD; *n* = 3. tGFP: turbo green fluorescent protein; BFP: blue fluorescent protein; huCD4: human CD4; SP: signal peptide; CADA: cyclotriazadisulfonamide; ERLEC1: endoplasmic reticulum lectin 1; PTK7: inactive tyrosine-protein kinase 7; HEK293T: human embryonic kidney 293T cells; DMSO: dimethyl sulfoxide; pPL: pre-prolactin; DNAJC3: DnaJ homolog subfamily C member 3; TMD, transmembrane domain.

The previous observation that a preprotein fraction could be rescued when cells are treated with a combination of CADA and MG132 indicates that an early event in protein translocation is blocked by CADA at a stage when the SP is not yet cleaved from the preprotein, and the protein is not yet glycosylated. Additionally, the limited but constant amount of rescued ERLEC1 preprotein measured in the CADA samples suggests that a saturating level of preprotein is reached, presumably corresponding to the fraction of preprotein that is stalled at the protected ribosome-bound translocon. Thus, it seems that CADA induces stalling of the preprotein at the translocon with subsequent extraction and further degradation of the respective preprotein by the proteasome. To explore if DNAJC3 contributes to the clearance of the CADA-stalled preproteins, we made use of fluorescently labelled CADA substrates in a tGFP-P2A-BFP backbone as described in a recent report [69]. This reporter construct encodes BFP downstream of a viral P2A sequence (Figure 5A), resulting in the transcription of polycistronic mRNA that is translated into two separated proteins in equal amounts, with cytosolic BFP serving as an internal protein translation control. By means of flow cytometry, the amount of tGFP and BFP was quantified for cells co-transfected with substrate-tGFP-P2A-BFP and either WT DNAJC3 or pPL-DNAJC3. In line with our previous report [69], the three substrates (i.e., WT huCD4, PTK7/huCD4 and ERLEC1/huCD4 chimaeric proteins) showed sensitivity to CADA in a concentration-dependent way (Figure 5B) which was similar to the sensitivity of full-length WT protein, as determined for PTK7 (Supplementary Figure S4A,B). The effect of CADA was related to the presence of an SP, given that an ERLEC1 mutant without an SP remained resistant to CADA (Figure 5B). As shown in Figure 5C, a detectable concentration-dependent decrease in BFP expression was observed when the substrates were treated with CADA (Figure 5C, black lines). Interestingly, the CADA-induced decrease in BFP could be nearly completely restored by co-transfecting the cells with the WT DNAJC3 plasmid, with a significant effect for ERLEC1 at the highest CADA concentrations (Figure 5C, orange lines). Furthermore, the reduction in BFP could be prevented (for PTK7) or even reversed (for huCD4 and ERLEC1) by pPL-DNAJC3 co-transfection, resulting in significantly enhanced BFP expression for ERLEC1 and huCD4 as compared to the non-co-transfected cells (Figure 5C, blue lines). Comparable results were obtained with the full-length PTK7 protein (Supplementary Figure S4C). Of note, the enhanced BFP levels under CADA pressure when cells expressed high levels of DNAJC3 could only be evoked by a CADA-sensitive substrate, given that a CADA-resistant construct of ERLEC1 (missing the SP) expressed cytosolic BFP at constant levels, irrespective of the DNAJC3 expression (Figure 5D). This indicates that CADA-treatment has no direct inhibitory effect on protein translation as such (i.e., ER-translocation independent), an effect that was also observed in a cell-free in vitro translation system [64]. Additionally, our BFP data show that when co-translational translocation of a substrate is blocked by CADA, translation of a downstream cytosolic protein is enhanced when DNAJC3 is present, suggesting that multiple attempts of protein translation from the same polycistronic transcript can be made once initially stalled proteins have been removed and blocked translocons have been cleared to restart protein translation.

**Figure 6:**
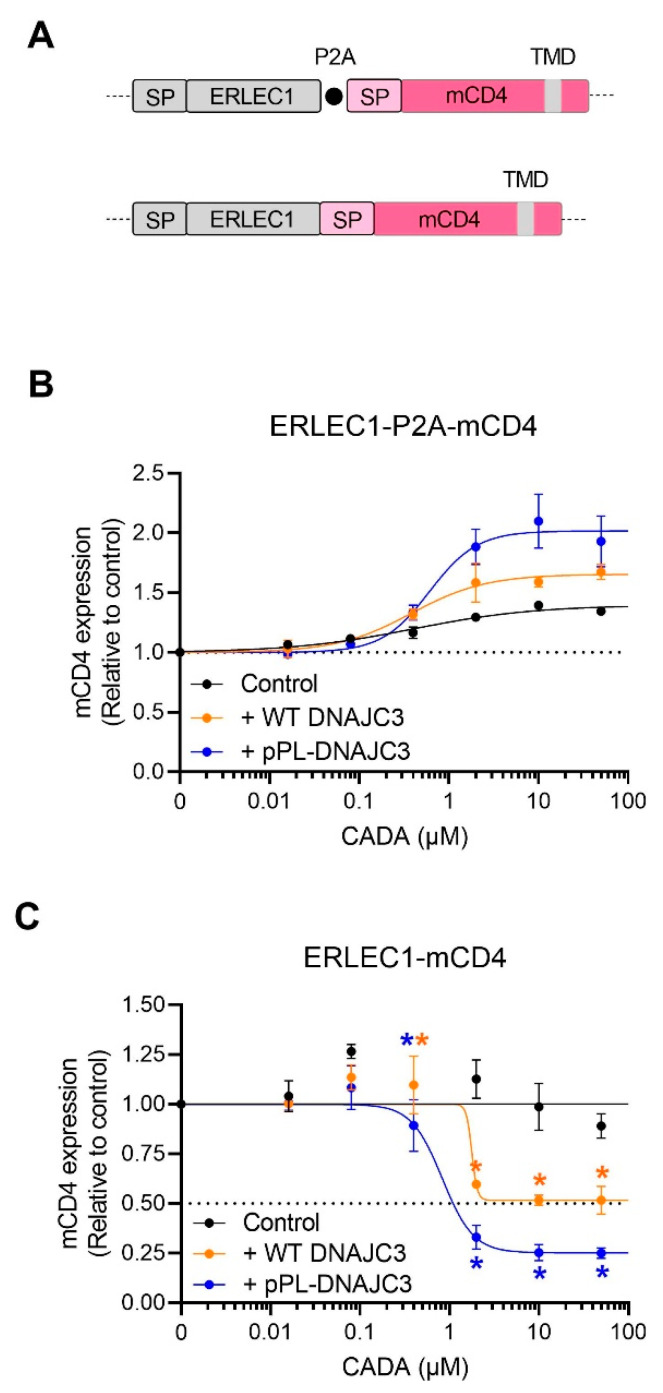
DNAJC3 affects the expression of a CADA-resistant protein differentially depending on the nature of the transcript. (**A**) Representation of the ERLEC1-mCD4 chimaeric constructs. The constructs expressed intracellular ERLEC1 and cell surface mCD4 that was anchored in the plasma membrane via its transmembrane domain. In the first construct, the two proteins were separated by a P2A sequence, whereas, in the second construct, the SP of mCD4 was directly fused to the C-terminus of ERLEC1. (**B**) Four-parameter concentration–response curves for the CADA of mCD4 that was cloned in the ERLEC1-P2A-mCD4 backbone as shown in (**A**). HEK293T cells were transiently transfected with ERLEC1-P2A-mCD4 plasmid DNA, or co-transfected with ERLEC1-P2A-mCD4 plasmid DNA and WT DNAJC3 or pPL-DNAJC3 and incubated with different CADA concentrations for 24 h. Transfected ERLEC1-P2A-mCD4 plasmid DNA was equal to the transfected ERLEC1-P2A-mCD4 plasmid DNA in the co-transfected conditions. Cells were fixed, permeabilized and stained with an anti-mCD4 antibody. Total levels of mCD4 in CADA-treated samples were normalised to the DMSO control (set at 1.0). Curves were fitted to data from two replicate experiments. Values are mean ± SD; *n* = 2. (**C**) Similar as in (**B**) but for the second construct without the P2A sequence. Curves were fitted to data from three replicate experiments. Values are mean ± SD; *n* = 3, except for the 0.08 µM CADA samples of control and WT DNAJC3 for which *n* = 2. Statistical analysis (multiple unpaired *t*-tests) showed significantly decreased expression of mCD4 when co-expressed with WT DNAJC3 and pPL-DNAJC3 as compared to the control. Of note, the values of 0.4 µM CADA for the ERLEC1-mCD4 construct only (black dots) have been removed to plot the black curve. These values are represented separately in Supplementary Figure S5. mCD4: mouse CD4; ERLEC1: endoplasmic reticulum lectin 1; CADA: cyclotriazadisulfonamide; HEK293T: human embryonic kidney 293T cells; pPL: pre-prolactin; DNAJC3: DnaJ homolog subfamily C member 3. (* = *p* < 0.05).

Finally, we analyzed the combined effect of CADA with high DNAJC3 levels on the expression of a CADA-resistant type I transmembrane protein (i.e., mouse CD4) that is expressed in cis of a CADA-sensitive substrate (see representation in Figure 6A). In this construct, mCD4 is separated from ERLEC1 by a P2A sequence, similar to the construct with BFP. Interestingly, without co-expression of DNAJC3, levels of mCD4 were enhanced under CADA pressure (Figure 6B, black line), suggesting that targeting of the mCD4 protein to the ER membrane might be more efficient when the upstream ERLEC1 protein is stalled at the translocon by CADA. In accordance with the BFP data, high levels of DNAJC3 enhanced the expression of mCD4 (Figure 6B, blue line), which could be explained by the DNAJC3 mediated accelerated clearance of blocked translocons and/or enhanced re-initiation of protein translation. Remarkably, when the mCD4 sequence (containing its own SP) was fused directly to the ERLEC1 sequence without the separation by P2A, mCD4 protein expression became regulated by CADA (Figure 6C, black line). Blocking the translocation of ERLEC1 by CADA also slowed down the translocation of the C-terminal part of the fusion protein. However, the presence of the CADA-resistant mCD4 SP preserved some translocation autonomy for mCD4. Of note, in the control samples without CADA the translation of mCD4 when directly fused to ERLEC1 (Figure 6C) was clearly less productive as compared to the construct that contained the P2A sequence (Figure 6B), resulting in mCD4 levels that represent only 2% of the P2A counterpart as determined by the flow cytometric mean fluorescence intensity (MFI) values (Supplementary Figure S5A). Remarkably, low concentrations of CADA (400 nM) enhanced mCD4 expression tremendously, again pointing to a stabilizing effect of CADA on the insertion of the preprotein in the translocon with higher targeting and translocation effect as a result (Supplementary Figure S5B). Co-expression of DNAJC3 significantly reduced the level of translocated mCD4 protein, which was most evident when the cells were co-transfected with the CADA-resistant pPL-DNAJC3 (Figure 6C, blue line). This would suggest that DNAJC3 quickly removes the stalled mCD4 protein from the Sec61 translocon, thereby reducing the putative attempts of the stalled protein from being transported into the ER lumen, thus, preventing the protein from ultimately being translocated under CADA pressure.

## 3. Discussion

Following up on a recent proteomics survey [69], in this study, we addressed the impact of altered DNAJC3 levels by small-molecule CADA on a few other CADA substrates, such as huCD4, ERLEC1 and PTK7. By comparison of a CADA-sensitive WT DNAJC3 with a CADA-resistant variant, we concluded that elevated DNAJC3 levels enhanced the inhibitory effect of CADA on the co-translational translocation of its substrates. Additional experiments with a proteasome inhibitor resulted in the rescue of a small fraction of preprotein under CADA pressure, pointing at a stalling effect of CADA on its substrates at the ribosome/Sec61 translocon complex. Finally, by means of flow cytometry, we demonstrated the DNAJC3-related enhanced protein translation of reporter proteins that were expressed downstream of a CADA-sensitive substrate. All these data together suggest a potential role of DNAJC3 in the clearance of clogged translocons by stalled ribosomes.

Although DNAJC3 has been validated as a substrate for CADA, our data confirm that DNAJC3 is not the most sensitive target for CADA. However, at high CADA concentrations, approximately 60% reduction in endogenous DNAJC3 and 75% decrease in transfected WT DNAJC3 was achieved that can clearly impact DNAJC3-related pathways. The inhibitory effect of CADA on the transfected WT DNAJC3 seemed to be stronger as compared to the endogenous protein (Figure 1F versus Figure 1C). This might be the result of the transfection protocol in which the compound is added as early as the DNAJC3 protein synthesis starts to prevent the biosynthesis of the protein, whereas, for endogenous DNAJC3, the net effect of CADA also relies on the natural turn-over (and degradation) of the existing cellular DNAJC3 source before the addition of CADA. Longer treatment (>24 h) with CADA generally enhances the protein down-modulating effect, as reported for huCD4 [62]. Regarding the cellular DNAJC3 protein level, given that (i) transfected cells express approximately 16 times more DNAJC3 as compared to the endogenous level (Supplementary Figure S1D), and (ii) high CADA-treatment still allows 25% expression of the protein (Figure 1F), different absolute expression levels of DNAJC3 were obtained under 10 µM CADA pressure, increasing gradually from a 0.4-fold, to 4-fold, to 16-fold increase for non-transfected, WT DNAJC3 and pPL-DNAJC3 transfected cells, respectively (as compared to the untreated non-transfected control). One should also interpret the obtained results (e.g., Figure 5) in light of these dose-response effects of CADA on DNAJC3.

One of the challenges of our study is the limited knowledge of DNAJC3, especially, about the different forms of the protein (with or without SP) and the cellular localisation (ER lumen or cytosolic). Our cell-free in vitro translation data clearly showed the existence of two different translocated species of DNAJC3 in the PK-protected ER lumen. The presence of a hydrophobic SP for one of the translocated DNAJC3 species suggests the anchoring of DNAJC3 in the ER membrane; however, additional experiments (e.g., alkaline flotation) are needed to verify this. The cell-free translation experiments were not performed with the WT DNAJC3 protein, though the DNAJC3/huCD4 chimaeric construct most likely represents a reliable alternative, given that it contains the N-terminal region of DNAJC3 sufficient to retain the targeting and gating features of WT DNAJC3 [72]. Accordingly, PK treatment of a DNAJC3/pPL chimaeric variant (used in a previous study [69]) also revealed a subfraction of uncleaved translocated DNAJC3 (Supplementary Figure S2). This indicates that the SP of DNAJC3 might contribute to the ultimate expression and subcellular localisation of the protein. Of course, most of our study is based on the comparison of WT DNAJC3 with a CADA-resistant pPL variant in which the SP of DNAJC3 has been exchanged. This could not only have an impact on sensitivity to CADA but also on the amount of ER membrane-anchored DNAJC3 species. From previous experiments with WT pPL (Supplementary Figure S2), we assume that SP cleavage of pPL-DNAJC3 should be complete and that solely SP-cleaved mature DNAJC3 proteins are present in the ER lumen, but this needs further investigation. Thus, depending on the role of the uncleaved WT DNAJC3 species, the comparison between CADA-sensitive and CADA-resistant DNAJC3 might not only be limited to the different levels of free DNAJC3 in the ER lumen under CADA pressure; therefore the presence or absence of the membrane-anchored DNAJC3 species should also be taken into account. However, in our immunoblot samples from HEK293T cells, we were not able to distinguish a putative uncleaved DNAJC3 variant, questioning the survival (and existence) of those species in cellulo. Furthermore, the significant differences in substrate expression between the WT DNAJC3 and pPL-DNAJC3 transfected cells (Figure 3) suggest that the SP-cleaved lumenal DNAJC3 protein is most likely the main driver in our study. Additionally, based on the consistent dose–response effects seen in Figure 5 and Figure 6, the contribution of a membrane-anchored DNAJC3 species in CADA activity is questionable, given that the condition with transfected WT DNAJC3 (orange curves in Figure 5 and Figure 6) would be the one with the highest amount of uncleaved translocated DNAJC3.

In the rested state, DNAJC3 is localised in the ER lumen where it has a multifunctional role in the protection of cells from the detrimental effects of ER stress. For instance, DNAJC3, alongside BiP, avoids protein misfolding in the early stages of ER stress after which it is involved in the upregulation of protein synthesis [32,33,35]. This way, DNAJC3 reassures the re-initiation of cellular processes post ER stress and, thus, contributes to the overall fitness of the cells [32,33,34]. The importance of DNAJC3 during ER stress has been evidenced in different independent clinical trial studies on the DNAJC3-related pathogenicity [51,52]. The DNAJC3-related pathogenicity involves a systematic phenotype, including the early onset of diabetes mellitus and different neurological disorders [39,50,51,52]. The latter is evidenced as a causal effect of the accumulation of cholesterol in the ER lumen of DNAJC3 deficient cells [39,53,54]. Interestingly, perturbed cholesterol metabolism is a reported pathophysiological observation in neurological diseases such as Alzheimer’s disease and Niemann–Pick type C disease [73,74].

A clear and consistent effect of CADA treatment is the preservation of preprotein species that could be visualized by immunoblotting. For ERLEC1, these preproteins could be identified even without inhibition of the proteasome. As ERLEC1 is a soluble protein of the ER lumen, the protein has no TMD that can escape the translocon laterally to be inserted in the lipid bilayer as is the case for huCD4 and PTK7. Additionally, ERLEC1 has no cytosolic tail that can be sensed by other cytosolic chaperones for proteasomal degradation. It is plausible that the stalled ERLEC1 preprotein resides in the protected channel of the ribosome exit tunnel and Sec61 translocon to escape proteasomal degradation. In contrast, for huCD4, only very limited amounts of preprotein could be seen, and only when rescued by MG132 treatment. It is known that the half-life of huCD4 is significantly lower in non-lymphoid cells (such as HEK293T) as compared to T cells because of the lack of the tyrosine kinase p56^lck^ that normally stably interacts with the cytosolic tail of huCD4 to prevent lysosomal degradation [75]. Thus, the degradation of the huCD4 preprotein might be more directed towards the lysosomal pathway, explaining the barely detectable amount of rescued preprotein by proteasome inhibition. The most obvious results with MG132 treatment were obtained for PTK7. As this is the least sensitive substrate of the three tested CADA targets, with the partial inhibition of PTK7 expression at the highest CADA concentrations (Figure 4B and Supplementary Figure S3B), we were able to detect additional inhibitory effects by DNAJC3 co-transfection and/or MG132 treatment. The synergistic effect of MG132 with CADA also suggested that with CADA some residual translocation of PTK7 was still occurring, and that this could proceed if the few clogged translocons were continuously cleared. Complete proteasome inhibition shuts down the clearance of blocked translocons, thus, preventing new rounds of PTK7-targeting and translocation, thus, reducing the expression of the mature proteins. In contrast to the two other CADA substrates, only for PTK7 could significantly enhanced levels of preprotein be measured when the cells were expressing high levels of DNAJC3. This would indicate that the lumenal DNAJC3 is involved in diverting the stalled PTK7 preprotein to the proteasome. Of course, we have no evidence of direct interaction between DNAJC3 and the stalled protein PTK7, and DNAJC3 might not only enhance the clearance of clogged translocons but might also have a positive effect on restarting protein translation for the next round of PTK7-targeting and SP insertion in an available translocon.

The data from the P2A-BFP constructs are indicative of the reduced translation of the C-terminal part of a stalled CADA substrate, suggesting that because of stalling by CADA, the ribosome does not ‘reach’ the very end of the transcript and, thus, cannot translate the BFP part. In this stalled situation, the ribosome may detach more frequently from the transcript at the P2A sequence. However, reduced translation is not because of a direct effect of CADA on the translation process as such, as proven by the ERLEC1 construct without the SP. Considering PTK7, co-transfection with pPL-DNAJC3 can restore the reduction in BFP levels to normal control levels, both for the PTK7/huCD4 chimaeric protein (Figure 5C) as for the WT full-length PTK7 control (Supplementary Figure S4C). In this context, DNAJC3 can help to rapidly clear the clogged translocons and to restart protein translation to normal levels. Unexpectedly, for ERLEC1 (and to a lesser content also huCD4), high levels of DNAJC3 seem to enhance even the translation of BFP under CADA pressure. This might be related to the strong inhibition of CADA on the translocation of these substrates, as compared to the partial inhibition on PTK7 (5-fold less sensitive). The stronger inhibition of protein translocation by CADA might induce a stronger ‘clogging signal’ to clear the translocon more rapidly, resulting in enhanced re-initiation of BFP translation, whereas, for PTK7, CADA might have a more decelerating effect on translocation and subsequent BFP production.

**Figure 7:**
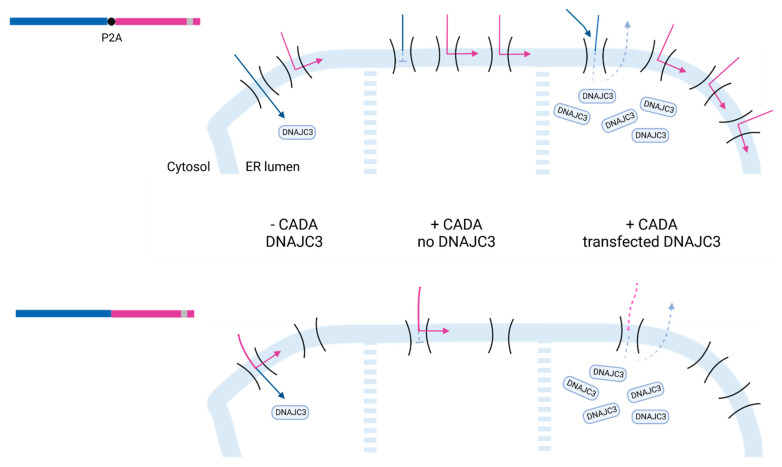
Proposed model of co-translational translocation events in the presence of CADA and DNAJC3. Top panel: The ERLEC1-P2A-mCD4 construct was translated by the ribosome and was targeted to the ER membrane with import into the ER lumen of the ERLEC1 protein (blue arrow) and insertion of mCD4 in the ER membrane (pink arrow). Under CADA pressure, ERLEC1 was prevented from being translocated (clogged translocon) while mCD4 could still get inserted into the ER membrane via its own SP, with even higher efficiency. When DNAJC3 was highly expressed, the ERLEC1 clogged translocon was rapidly cleared, allowing insertion of more mCD4 in the ER membrane while ERLEC1 translocation was consistently inhibited by CADA. Bottom panel: The ERLEC1-mCD4 construct was translated but the ER targeting and membrane insertion of mCD4 was at a very low level. Treatment with suboptimal levels of CADA stabilized the targeting complex and enhanced the targeting and membrane insertion of mCD4, whereas, at high CADA concentration, the translation and translocation of mCD4 were slightly down-regulated. The presence of high DNAJC3 levels rapidly removed (and degraded) the stalled ERLEC1 species (and downstream mCD4 protein) and prevented the stalled mCD4 from eventually getting inserted into the ER membrane, thus, intensifying the net down-modulating effect of CADA on its substrates.

The most exciting data were obtained with the ERLEC1-mCD4 constructs (Figure 6). Here, the presence of the P2A sequence clearly impacted the outcome of mCD4 expression in relation to DNAJC3 levels (Figure 7). In general, exchanging cytosolic BFP by an ER-targeting mCD4 did not change the relative order of the reporter expression level in relation to the different DNAJC3 constructs (with pPL-DNAJC3 co-transfection giving the highest upregulation). However, without exogenous DNAJC3, the presence of CADA enhanced even the expression of mCD4. Here, one of the most suitable explanations would be that of the enhanced targeting efficiency of mCD4 because of a stalled ribosome that had already docked onto the ER membrane and was slowed down in its translation. This would give mCD4 more time and higher chances to insert in a translocon quickly and successfully, thus, enhancing the expression levels of mCD4. The additional upregulating effects on mCD4 expression by DNAJC3 could then be because of more cleared translocons that would be available in the vicinity of the stalled N-terminal ERLEC1. In contrast, the expression of an mCD4 protein directly fused to ERLEC1 would become regulated and suppressed by CADA. This is not unexpected, given that the mCD4 protein is in fact an elongated ERLEC1 variant that is mainly controlled by the CADA-sensitive SP of ERLEC1. However, the rather limited CADA sensitivity of the mCD4 part in the control cells transfected with ERLEC1-mCD4 suggests that the expression of mCD4 was still under the control of its own (CADA-resistant) SP. Additional research is needed to further explore the functionality of the mCD4 SP in this context. The very low expression efficiency of this mCD4 fusion protein as compared to the P2A variant (Supplementary Figure S5A) might be related to the unsuccessful ER translocation of mCD4 and putative altered topology with a mainly cytosolic expression of the extracellular CD4 region that did not survive the cytosolic degradation machinery (the SP might have served as a kind of TMD in the head-on insertion without SP cleavage). Nevertheless, the low translocation speed of mCD4 (due to the stalled ERLEC1 part) might result in higher residual translocation into the ER lumen due to the longer dwelling time of the temporarily stalled substrate in the translocon. The low translocation efficiency of this construct magnified the impact of altered DNAJC3 levels in this process. Quickly removing the stalled targets by DNAJC3 considerably reduced the dwelling time of cargo in the translocon and prevented the residual ‘slipping’ of the preprotein into the ER lumen. Thus, by directly reducing DNAJC3 protein levels, CADA attenuates its net down-modulating effect on the expression of affected targets.

In conclusion, the present study on the relation between CADA and DNAJC3 revealed that higher cellular levels of DNAJC3 enhanced the sensitivity of huCD4, PTK7 and ERLEC1 for CADA. It also showed that CADA treatment resulted in the preservation of a small preprotein fraction, most likely corresponding with stalled CADA substrates at the ribosome/Sec61 translocon complex. DNAJC3 positively affected protein translation of a reporter protein expressed downstream of a CADA-stalled substrate, suggesting that DNAJC3 promotes the clearance of the clogged translocon. Altogether, we hypothesize that reduced DNAJC3 levels by CADA treatment retards the clearance of clogged Sec61 translocons filled with CADA-stalled substrates. This might allow more residual translocation of CADA substrates into the ER lumen due to the longer dwelling time of the temporarily stalled substrates in the translocon. Thus, by directly reducing DNAJC3 protein levels, CADA attenuates its net down-modulating effect on its substrates which can be related to the generally low impact of CADA on the proteome. However, further knowledge and research on DNAJC3-related pathways in ER stress would be of great help to improve our understanding of the cellular mechanism behind blocked co-translational translocation events, and the impact of small molecule translocation inhibitors at the molecular level.

## 4. Materials and Methods

### 4.1. Compounds and Antibodies

CADA hydrochloride was synthesized as described previously [76]. CADA was dissolved in dimethyl sulfoxide (DMSO) and stored at a stock concentration of 10 mM at room temperature. Western blot and flow cytometry antibodies were purchased from (i) Genscript (Piscataway, NJ, USA): anti-V5 (cat #A01724); (ii) BD Biosciences (Allschwil, Switzerland): anti-clathrin (cat. #610500); (iii) Thermo Fisher Scientific (Waltham, MA, USA): anti-β-actin (cat. #MA1-140) and allophycocyanin (APC)-labelled anti-mouse CD4 (clone GK1.5; cat. #47-0041-82); (iv) Sigma (Saint Louis, MO, USA): anti-FLAG (cat. #F1804); (v) Cell Signaling Technology (Danvers, MA, USA): anti-DNAJC3 (p58IPK) (cat. #2940S); (vi) Dako (Santa Clara, CA, USA): HRP-labelled goat anti-mouse immunoglobulins (cat. #P0447) and HRP-labelled swine anti-rabbit (cat. #P0399).

### 4.2. Plasmids and Mutagenesis

As described in a previous report [69], the ERLEC1 expression vector (pGEM-T backbone) was purchased from Sino biological (Beijing, China), the PTK7 expression vector (pDONR223 backbone) from Addgene (Watertown, MA, USA) and the DNAJC3 expression vector (pDONR223 backbone) from the DNASU plasmid repository (Tempe, AZ, USA).

Constructs for western blot were designed to include the simian virus 5 (V5) epitope (GKPIPNPLLGLD) at the C-terminus of the protein of interest. Site-directed mutagenesis of all constructs was performed with the Q5 site-directed mutagenesis kit (New England Biolabs, Ipswich, MA, USA) or NEBuilder HiFi DNA assembly kit (New England Biolabs, Ipswich, MA, USA) following the manufacturer’s instructions. Plasmid DNA was isolated using the Nucleospin Plasmid Transfection grade system (Macherey Nagel, Düren, Germany) supplemented with an endotoxin removal wash. The concentration of all constructs was determined with a NanoDrop 1000 spectrophotometer and sequences were confirmed by automated capillary Sanger sequencing (Macrogen Europe).

### 4.3. Transient Transfection

HEK293T cells were cultured in Dulbecco’s modified eagle medium (DMEM), supplemented with 10% (*v*/*v*) fetal bovine serum (HyClone, Logan, UT, USA). Cells were seeded at 4 × 10^5^ cells/mL and incubated overnight at 37 °C prior to transfection the next day. Lipofectamine LTX (Thermo Fisher Scientific, Waltham, MA, USA) was used for the transfection of plasmid DNA according to the manufacturer’s protocol. CADA and/or MG132 (Sigma, Saint Louis, MO, USA) was added 6 h post transfection and cells were lysed for immunoblotting or fixed in paraformaldehyde for antibody staining and subsequent flow cytometric analysis.

### 4.4. Immunoblotting

After CADA treatment, cells were collected and lysed in Nonidet P-40 buffer (1%, supplemented with 50 mM Tris HCl, pH 8.0, 150 mM NaCl, protease inhibitor cocktail (Roche, Basel, Switzerland) and PMSF). Lysates were run on between 4 and 12% Criterion XT Bis-Tris gels in MES buffer (Bio-Rad, Hercules, CA, USA), transferred to PVDF or nitrocellulose membranes using the BioRad Trans-Blot Turbo transfer system (Bio-Rad, Hercules, CA, USA), blocked with 5% non-fat dried milk in TBST and incubated with a primary and secondary antibody. SuperSignal West Pico and Femto chemiluminescence reagent (Thermo Fisher scientific, Waltham, MA, USA) was used for detection with a ChemiDoc MP system (Bio-Rad, Hercules, CA, USA). Signal intensities were quantified with Image Lab software v5.0 (Bio-Rad, Hercules, CA, USA). Differences in protein concentration between each lane were compensated by normalisation to the clathrin heavy chain or β-actin signal. To compare the down-modulating activity of CADA, IC50 values were calculated with GraphPad Prism 8 software (San Diego, CA, USA) on a four-parameter concentration–response curve fitted to data from at least three replicate experiments with flow cytometry. The absolute IC50 value represented the compound concentration that resulted in the 50% reduction in the protein level.

### 4.5. Antibody Staining and Flow Cytometry

After CADA treatment, cells were collected, fixed and permeabilized with fixation/permeabilization solution (BD biosciences, Allschwil, Switzerland) and subsequently washed with perm/wash solution (BD biosciences, Allschwil, Switzerland). Next, cells were stained with antibodies and incubated at 4 °C for 45 min. Samples were then washed with perm/wash solution (BD biosciences) and fixed in PBS containing 1% formaldehyde before acquisition on BD FACS Celesta flow cytometer (Beckton Dickinson) with BD FACSDiva 8.0.1 software (BD biosciences, Allschwil, Switzerland). All data were analyzed in FlowJo X v10 (BD biosciences, Allschwil, Switzerland).

### 4.6. Cell-Free In Vitro Translation and Translocation

The DNAJC3 SP and first 62AA of mature DNAJC3 were fused upstream of huCD4 with PCR. The Qiagen EasyXpress linear template kit was used to generate linear DNA fragments using PCR. The PCR products were purified and transcribed in vitro using T7 RNA polymerase (RiboMAX system; Promega, Madison, WI, USA). All transcripts were translated in the presence of rabbit reticulocyte lysate (Promega, Madison, WI, USA) in the presence of L-35S- methionine (PerkinElmer, Waltham, MA, USA). Translations were performed at 30 °C in the presence or absence of ovine pancreatic microsomes and CADA as described elsewhere. Samples were treated with proteinase K (Roche, Basel, Switzerland) on ice for 30 min. Protein digestion was stopped with phenylmethylsulfonyl fluoride (PMSF; Thermo Fisher Scientific, Waltham, MA, USA) after which samples were washed with a low-salt buffer (80 mM KOAc, 2 mM Mg(OAc)_2_, 50 mM Hepes, pH 7.6), and radiolabelled proteins were isolated by centrifugation (10 min at 21.382 g, 4 °C). The proteins were then separated with SDS-PAGE and detected by phosphor imaging (Cyclone Plus storage phosphor system; PerkinElmer, Waltham, MA, USA).

## Supplementary Materials

The following supporting information can be downloaded at: https://www.mdpi.com/article/10.3390/ijms23020584/s1.
