# Supplementary material for: Reduced DNAJC3 Expression Affects Protein Translocation across the ER Membrane and Attenuates the Down-Modulating Effect of the Translocation Inhibitor Cyclotriazadisulfonamide
Source: Int J Mol Sci. 2022 Jan 6;23(2):584. doi: 10.3390/ijms23020584 (PMC8775681; doi:10.3390/ijms23020584)
Supplement: Supplementary file 1 [file ijms-23-00584-s001.zip › ijms-1534273-supplementary.pdf]

SUPPLEMENTARY FIGURES

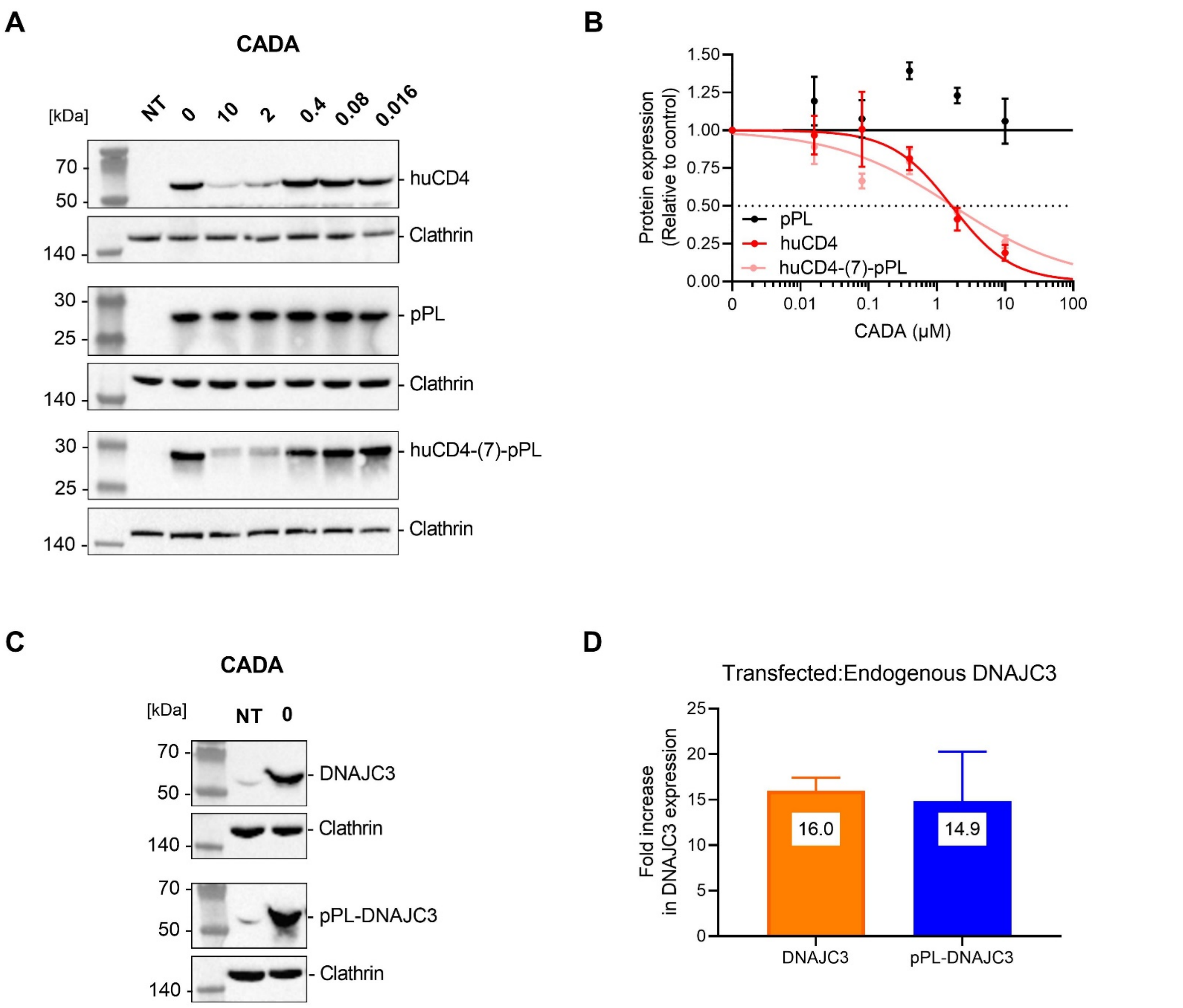

Figure S1. A) Western blot images of cell lysates from non-transfected (NT) and huCD4-FLAG, pPL-FLAG or huCD4-(7)-pPL-FLAG transfected HEK293T cells treated for 24h with different CADA concentrations. Protein bands were visualized with an antibody against the FLAG tag and an anti-clathrin antibody was used for the cell loading control. One representative experiment out of three or four experiments is shown. **B)** Concentration response curves of CADA for pPL, huCD4 and huCD4(7)-pPL transfected HEK293T cells. Protein bands from (A) were quantified and normalized to the clathrin internal control. A four-parameter concentration response curve was fitted to the data from at least two replicate experiments. Values are mean ± SD; n ≥ 2. **C)** Western blot images of cell lysates from non-transfected (NT) and DNAJC3 or pPL-DNAJC3 transfected HEK293T cells. Protein bands were visualized with an antibody against DNAJC3 and anti-clathrin antibody was used for the cell loading control. One representative experiment out of three is shown. **D)** Fold increase of transfected DNAJC3 and pPL-DNAJC3 expression compared to non-transfected control (endogenous DNAJC3 expression). Protein bands are quantified from (C). Bars are mean ± SE; n ≥ 2. Mean values are indicated in the white inserts. huCD4: human CD4, pPL: pre-prolactin, HEK293T: human embryonic kidney 293T cells, CADA: cyclotriazadisulfonamide, pPL: pre-prolactin, DNAJC3: DnaJ homolog subfamily C member 3.

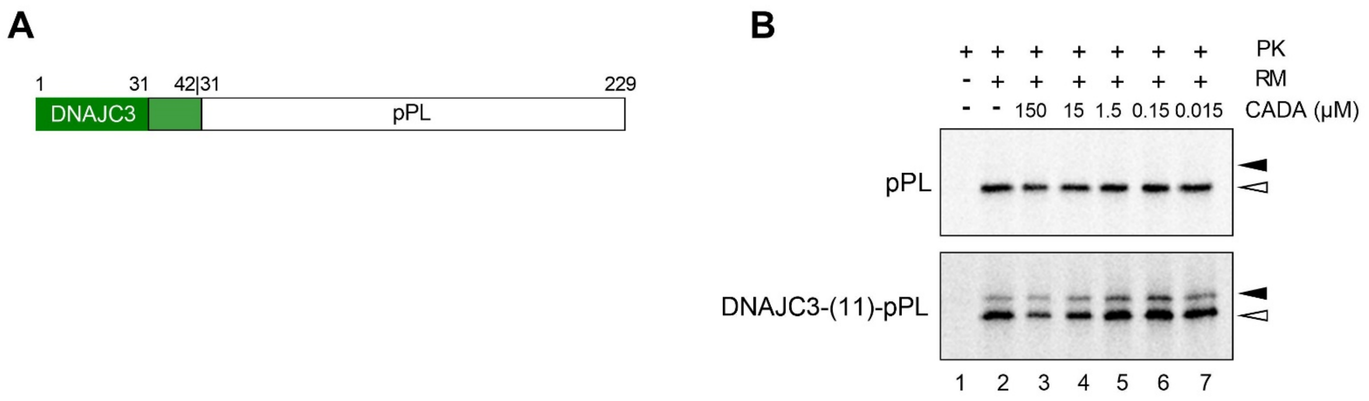

Figure S2. Cell-free *in vitro* translation of a DNAJC3-pPL chimaeric construct. **A)** Representation of the construct used for *in vitro* translation and translocation assay. **B)** Cell-free *in vitro* translation and translocation in rabbit reticulocyte lysate supplemented with ovine microsomes, CADA and proteinase K (PK). Autoradiogram of the *in vitro* translated and translocated pPL and DNAJC3-pPL chimaeric protein. In the presence of rough microsomes (RM), the preprotein (black arrowhead) is translocated into the ER lumen and the SP is cleaved, resulting in a faster migrating mature protein (open arrowhead). One representative experiment out of two is shown. CADA: cyclytriazadisulfonamide, DNAJC3: DnaJ homolog subfamily C member 3, PK: Proteinase K, RM: rough microsomes, ER: endoplasmic reticulum, SP: signal peptide.

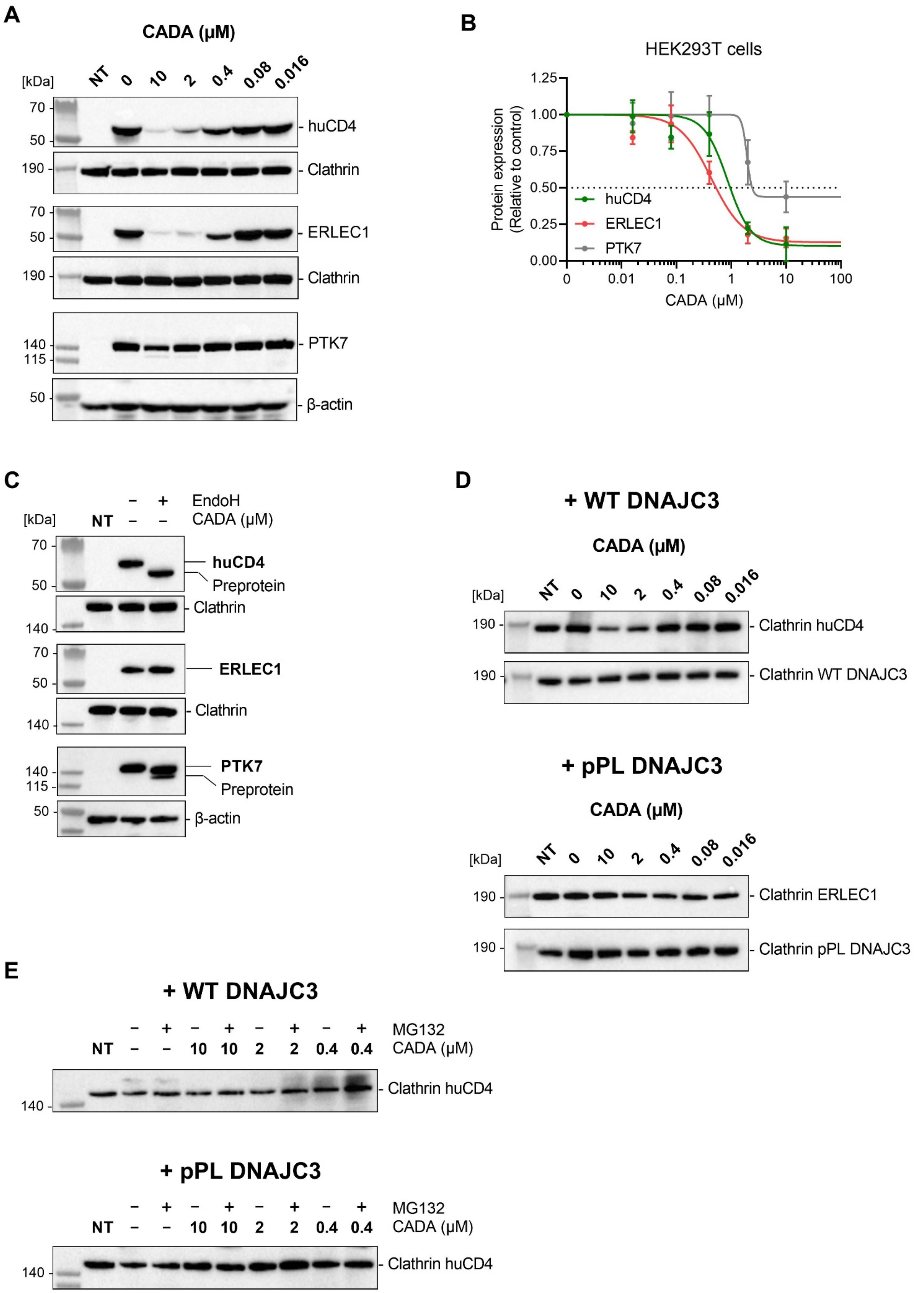

A
CADA (µM)
[kDa]
NT
0
10
2
0.4
0.08
0.016
70
50
huCD4
190
Clathrin
70
50
ERLEC1
190
Clathrin
140
115
PTK7
50
β-actin
B
HEK293T cells
Protein expression (Relative to control)
1.25
1.00
0.75
0.50
0.25
0.00
huCD4
ERLEC1
PTK7
0
0.01
0.1
1
10
100
CADA (µM)
C
EndoH
CADA (µM)
[kDa]
NT
70
50
huCD4
Preprotein
Clathrin
140
70
50
ERLEC1
Clathrin
140
140
115
PTK7
Preprotein
50
β-actin
D
+ WT DNAJC3
CADA (µM)
[kDa]
NT
0
10
2
0.4
0.08
0.016
190
Clathrin huCD4
190
Clathrin WT DNAJC3
+ pPL DNAJC3
CADA (µM)
[kDa]
NT
0
10
2
0.4
0.08
0.016
190
Clathrin ERLEC1
190
Clathrin pPL DNAJC3
E
+ WT DNAJC3
MG132
CADA (µM)
NT
10
10
2
2
0.4
0.4
Clathrin huCD4
140
+ pPL DNAJC3
MG132
CADA (µM)
NT
10
10
2
2
0.4
0.4
Clathrin huCD4
140

Figure S3. **A)** Western blot images of cell lysates from non-transfected (NT) and huCD4-V5, ERLEC1-V5 or PTK7-V5 transfected HEK293T cells treated for 24h with different CADA concentrations. The transfection was performed without co-transfection with the DNAJC3 constructs. Transfected huCD4, ERLEC1 and PTK7 plasmid DNA was equal to the transfected huCD4, ERLEC1 and PTK7 plasmid DNA in the co-transfected conditions of figures 3A and 3B. Protein bands were visualized with an antibody against the V5 tag, and an antibody against clathrin (huCD4 and ERLEC1) or β-actin (PTK7) was used for the cell loading controls. One representative experiment out of three to five experiments is shown. **B)** Concentration response curves of CADA for huCD4, ERLEC1 or PTK7 transfected HEK293T cells. Samples from (A) were quantified and normalized to the clathrin (huCD4 and ERLEC1) or β-actin (PTK7) internal control. A four-parameter concentration response curve was fitted to the data from at least two replicate experiments. Values are mean ± SD; $n \geq 2$. **C)** Western blot images of cell lysates from non-transfected (NT) and huCD4-V5, ERLEC1-V5 or PTK7-V5 transfected HEK293T cells. Total transfected huCD4, ERLEC1 and PTK7 was equal to the co-transfected conditions of figures 3A and 3B. Cell lysates were treated with Endo H prior to SDS-PAGE and western blotting to remove N-linked glycosylations. huCD4 is completely deglycosylated by Endo H, ERLEC1 revealed not to be glycosylated in HEK293T cells, whereas PTK7 showed a highly glycosylated protein, that is partially resistant to Endo H treatment, resulting in only a minor fraction of the glycoprotein that is deglycosylated by Endo H treatment. **D)** Western blot images of the additional clathrin loading controls of Figure 3. **E)** Western blot images of the additional clathrin loading controls of Figure 4. huCD4: human CD4, ERLEC1: endoplasmic reticulum lectin 1, PTK7: inactive tyrosine-protein kinase 7, HEK293T: human embryonic kidney 293T cells, CADA: cyclotriazadisulfonamide.

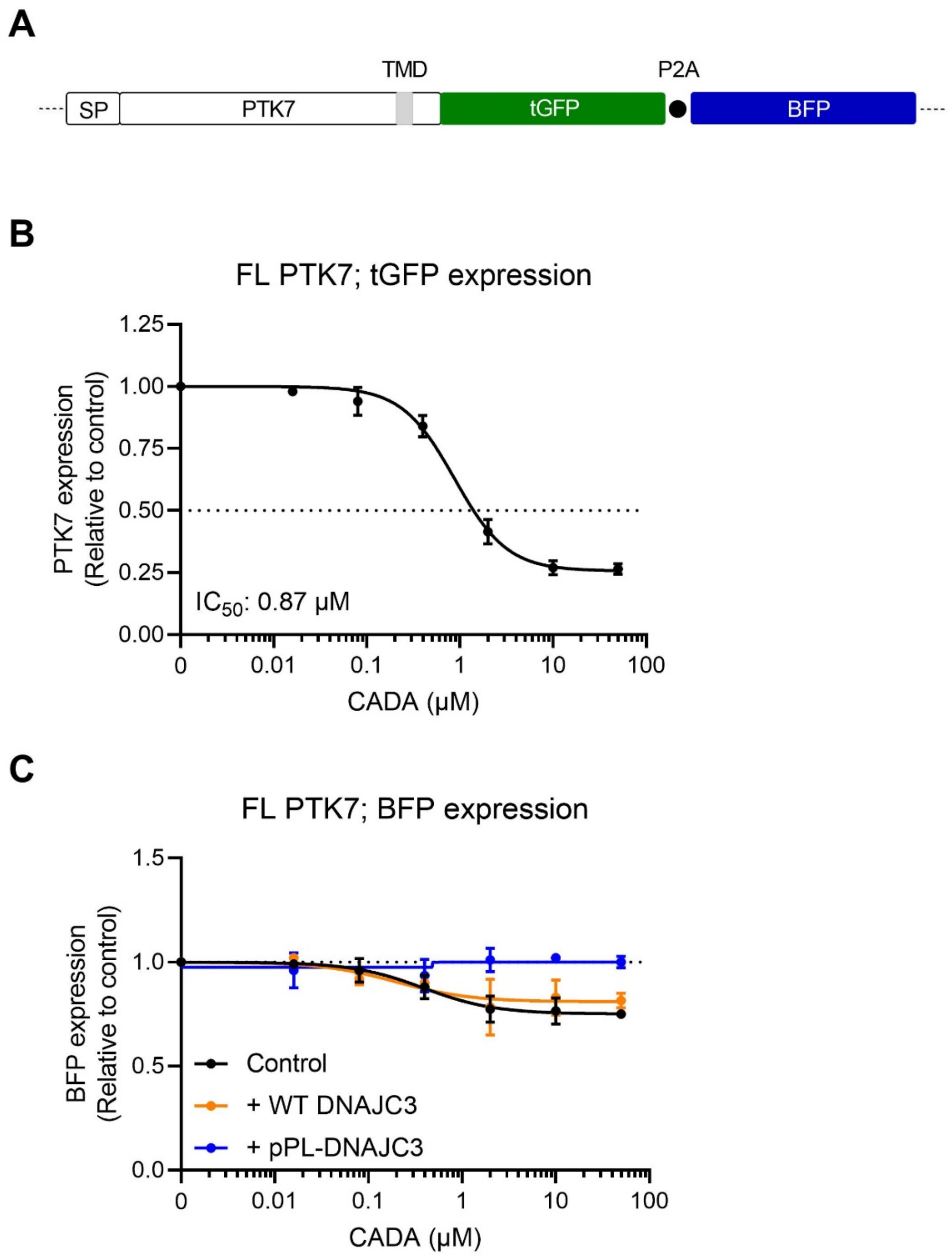

Figure S4. A) Representation of the PTK7-tGFP-P2A-BFP construct. The construct expresses full length wild-type PTK7 that is anchored in the plasma membrane via its transmembrane domain and with tGFP at the cytosolic tail. **B)** Four-parameter concentration response curve for CADA of PTK7 cloned in the tGFP-P2A-BFP plasmid backbone as shown in (A). HEK293T cells were transiently transfected with the PTK7-tGFP-P2A-BFP construct and incubated with different CADA concentrations for 24h. The total transfected PTK7-tGFP-P2A-BFP DNA was equal to the co-transfected conditions of (C). Protein levels in CADA treated samples are normalized to the DMSO control (set at 1.00). Curve is fitted to data from two replicate experiments. Values are mean ± SD; n = 2. **C)** Four-parameter concentration response curves for CADA of PTK7 cloned in the tGFP-P2A-BFP plasmid backbone as shown in (A). HEK293T cells were transiently co-transfected with the PTK7-tGFP-P2A-BFP construct and WT DNAJC3 or pPL-DNAJC3 and incubated with different CADA concentrations for 24h. BFP levels in CADA-treated samples are normalized to the DMSO control (set at 1.0). Curves are fitted to data from two replicate experiments. Values are mean ± SD; n = 2. tGFP: turbo green fluorescent protein, BFP: blue fluorescent protein, PTK7: inactive tyrosine-protein kinase 7, CADA: cyclotriazadisulfonamide, HEK293T: human embryonic kidney 293T cells, DMSO: dimethyl sulfoxide.

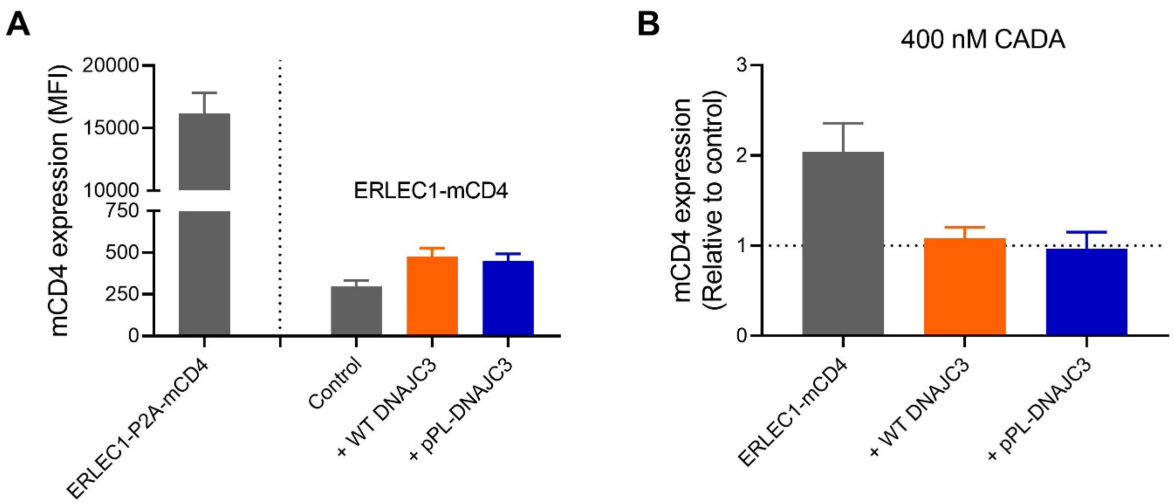

Figure S5. See legend to Figure 6B and 6C. (A) Mean fluorescence intensity (MFI) values for mCD4 staining of HEK293T cells transfected with the ERLEC1-P2A-mCD4 (left bar) or the ERLEC1-mCD4 construct (right part). The measurement was done on the non-treated (0 µM CADA) control samples, that were either non-transfected (= control; grey bars) or transfected with the indicated construct (colored bars). Do note that the expression of the ERLEC1-mCD4 control is only 2% of that of the ERLEC1-P2A-mCD4 control. Data were collected from at least 10 000 cells, with biological repeats. Bars are mean +/- SD; n ≥ 4. (B) Quantification of the mCD4 expression for the different constructs at 0.4 µM CADA treatment, relative to the respective untreated control. Do note a tremendous increase in mCD4 expression in the condition without DNAJC3 co-expression (grey bar).
